# Acellular Pertussis Booster in Adolescents Induces Th1 and Memory CD8^+^ T Cell Immune Response

**DOI:** 10.1371/journal.pone.0017271

**Published:** 2011-03-08

**Authors:** Nikolaus Rieber, Anna Graf, Dominik Hartl, Simon Urschel, Bernd H. Belohradsky, Johannes Liese

**Affiliations:** 1 University Children's Hospital, Ludwig-Maximilians-University, Munich, Germany; 2 University Children's Hospital, Eberhard-Karls-University, Tuebingen, Germany; 3 University Children's Hospital, Julius-Maximilians-University, Wuerzburg, Germany; University of Alabama-Birmingham, United States of America

## Abstract

In a number of countries, whole cell pertussis vaccines (wcP) were replaced by acellular vaccines (aP) due to an improved reactogenicity profile. Pertussis immunization leads to specific antibody production with the help of CD4^+^ T cells. In earlier studies in infants and young children, wcP vaccines selectively induced a Th1 dominated immune response, whereas aP vaccines led to a Th2 biased response. To obtain data on Th1 or Th2 dominance of the immune response in adolescents receiving an aP booster immunization after a wcP or aP primary immunization, we analyzed the concentration of Th1 (IL-2, TNF-α, INF-γ) and Th2 (IL-4, IL-5, IL-10) cytokines in supernatants of lymphocyte cultures specifically stimulated with pertussis antigens. We also investigated the presence of cytotoxic T cell responses against the facultative intracellular bacterium *Bordetella pertussis* by quantifying pertussis-specific CD8^+^ T cell activation following the aP booster immunization. Here we show that the adolescent aP booster vaccination predominantly leads to a Th1 immune response based on IFNgamma secretion upon stimulation with pertussis antigen, irrespective of a prior whole cell or acellular primary vaccination. The vaccination also induces an increase in peripheral CD8^+^CD69^+^ activated pertussis-specific memory T cells four weeks after vaccination. The Th1 bias of this immune response could play a role for the decreased local reactogenicity of this adolescent aP booster immunization when compared to the preceding childhood acellular pertussis booster. Pertussis-specific CD8^+^ memory T cells may contribute to protection against clinical pertussis.

## Introduction

*Bordetella pertussis* is a facultative intracellular bacterium associated with respiratory tract infections which may become life threatening especially in infants. In 2003, the WHO reported an estimated 17.6 million cases worldwide, resulting in 279.000 pertussis related deaths [1]. Young adults are the main source for transmission of pertussis to young unvaccinated infants [2]–[4]. For this reason, pertussis booster vaccinations for all adolescents, for all adults, or for adults with close contact to infants, are recommended in a number of countries, including the USA, Germany, France and Italy.

For many years only whole cell pertussis vaccines (wcP) were available. However, a considerable rate of systemic adverse effects were associated with their use [5]. Therefore, in a number of countries, wcP vaccines were replaced by acellular vaccines (aP) in the mid 1990s because of their improved reactogenicity profile [5]. The immune response induced by wcP and aP vaccines is different: wcP vaccines, like natural pertussis infections, selectively induce Th1 cells, characterized by a cytokine pattern consisting mainly of IL-2, TNF-α, and INF-γ; aP vaccines lead to a Th2-polarized cytokine pattern (mainly IL-4, IL-5) in infants and children at 4-6 years of age [6]-[9]. Importantly, when investigating long-term immune responses, these different effects may be masked by subclinical pertussis infections, which act as silent, natural boosters. In aP-vaccinated children, these silent boosters may cause a switch to the Th1 predominant phenotype [10].

We have recently shown that adolescents receiving an aP booster immunization are able to initiate substantial lymphoproliferative, cell mediated immunity (CMI) to pertussis antigens, not only when the initial immunization was wcP, but also with an exclusively acellular vaccination regimen [11]. In this study, we determined whether the distinct T helper cell responses normally seen following wcP versus aP preimmunization still exists after the delivery of an aP booster immunization in adolescents. Presuming an additive role for a cytotoxic T cell response against the facultative intracellular bacterium *B. pertussis* we also investigated pertussis-specific CD8^+^ T cell activation following vaccination with an aP booster.

Our study population consisted of three groups of adolescents who had received either 4 wcP, 4aP or 5aP doses during infancy and early childhood. The participants with 5 aP doses belong to the first cohort of adolescents to have received 5 consecutive aP vaccinations in childhood before their adolescent aP booster vaccination. This schedule is now generally recommended in several countries, including the USA, France and Germany.

## Materials and Methods

### Ethics statement

The study protocol was approved by the local ethical review board of the University of Munich, Germany, and by the Paul-Ehrlich-Institute, Langen, Germany. The study was conducted according to Good Clinical Practice and in accordance with the Declaration of Helsinki. Written, informed assent from each participant and written, informed consent from the parent/legal guardian was obtained prior to study entry.

### Study population

This open multicenter study was carried out to examine pertussis-specific T cell immune responses before and after aP booster vaccination in adolescents with different childhood pertussis vaccination schedules. Patients were recruited from pediatric practices in Germany and in the outpatient department of the University Children's Hospital, Ludwig-Maximilians-University, Munich.

A clinical trial examining reactogenicity and immunogenicity of a Tdap or Tdap plus polio vaccination given as the 6^th^ consecutive acellular pertussis dose in 10-14 year old adolescents in Germany was conducted in parallel with the present study [11], [12]. Study investigators of the clinical trial were asked to participate as investigators in this study. Adolescents 10–14 years of age with no obvious health problems and no immunosuppressive treatment were enrolled, provided they had previously received either 5 doses of an acellular pertussis vaccine (Biken DTaP, last dose age 4 to 6 years) [13], [14] (*5aP group*), 4 doses of an acellular pertussis vaccine (Biken DTaP, last dose age 18 to 24 months) [13] (*4aP group*), or 4 doses of a whole-cell pertussis vaccine (DPT-Impfstoff®, Behringwerke, Marburg, Germany, last dose age 18 to 24 months) (*4wcP group*). The Biken DTaP was a sterile preparation of a two component acellular pertussis vaccine with pertussis toxin (PT) and filamentous hemagglutinin (FHA) manufactured by Biken and Tanabe Corp. (Osaka, Japan) combined with adsorbed diphtheria and tetanus toxoids by Aventis Pasteur Inc. (Swiftwater, PA). Each 0.5-ml dose contained 6.7 limes flocculation units (Lf) of diphtheria toxoid, 5.0 Lf of tetanus toxoid, 23.4 µg of detoxified PT and 23.4 µg of detoxified FHA, thimerosal 1/10 000 (25 µg), sodium phosphate and sodium chloride. The whole-cell pertussis vaccine DPT-Impfstoff® consisted of 4 IU of *B. pertussis*, 30 IU of diphtheria toxoid, 40 IU of tetanus toxoid and 0.75 mg of aluminum hydroxide/aluminum phosphate and was used as comparative whole-cell vaccine in an acellular pertussis vaccine efficacy study in Germany [13]. At the time of the study, an adolescent booster vaccination against pertussis, diphtheria and tetanus was recommended in Germany for all participants. No participants with a history of clinical pertussis disease were enrolled in the study.

### Study vaccine

Only licensed products provided by Sanofi Pasteur MSD, Leimen, Germany were used. Participants received either REPEVAX®, COVAXIS®, or COVAXIS® and IPV MERIEUX®.

COVAXIS® contains in each 0.5-ml dose 2.5 µg pertussis toxoid (PT), 5 µg filamentous hemagglutinin (FHA), 3 µg pertactin (PRN), 5 µg fimbriae types 2 and 3 (FIM), 2 Lf diphtheria toxoid, 5 Lf tetanus toxoid, 1.5 mg aluminum phosphate and 0.6% 2-phenoxyethanol. COVAXIS® and IPV-Merieux® are identical to the US-licensed products ADACEL® and IPOL®, respectively. REPEVAX®, the combination of the two, is not marketed in North America.

In addition to the same acellular pertussis antigens and content, each 0.5-ml dose of REPEVAX® additionally contains inactivated, Vero cell-derived poliomyelitis vaccine (vero cell origin) (Poliovirus Type 1: 40 D units, Poliovirus Type 2: 8 D units, Poliovirus Type 3: 32 D units).

IPV MERIEUX® contains in each 0.5-ml dose inactivated, Vero cell-derived poliomyelitis vaccine (vero cell origin) (Poliovirus Type 1: 40 D units, Poliovirus Type 2: 8 D units, Poliovirus Type 3: 32 D units).

### Study procedures

Participants of the *5aP group* and the *4wcP group* received 1 dose of REPEVAX vaccine in the left arm, or 1 dose of COVAXIS vaccine in the left arm plus 1 optional dose of IPV MERIEUX vaccine in the right arm, as determined by their randomisation in the parallel clinical reactogenicity trial [12].

*4aP group* participants received 1 dose of REPEVAX vaccine in the left arm, or 1 dose of COVAXIS vaccine in the left arm plus one optional dose of IPV MERIEUX vaccine in the right arm as recommended by their physician.

From every participant, a 10-ml heparinized blood sample (20 IU heparin per ml) and a 3-ml serum sample were drawn directly before vaccination and 28–36 days after vaccination. The blood samples were sent at room temperature to the Laboratory of Immunology at the University Children's Hospital, Ludwig-Maximilians-University, Munich, Germany and were processed within 24 hours.

### Preparation of peripheral blood mononuclear cells (PBMC)

PBMC were prepared by Ficoll density gradient sedimentation (Lymphocyte Separation Medium, Biochrom, Berlin, Germany) and washed twice in RPMI 1640 (Biochrom, Berlin, Germany). Trypan blue staining solution at 0.5% differentiated between viable and nonviable cells. Only samples with vitality of >80% were evaluated. PBMC were resuspended at 1.5×10^6^ cells/ml in RPMI 1640 supplemented with 5% heat-inactivated (30 min at 56°C) human serum and 1% penicillin (Biochrom, Berlin, Germany), hereafter referred to as complete medium.

### Lymphocyte stimulation

PBMC (1.5×10^5^) were cultured in round-bottom microtitre plates (Greiner bio-one, Frickenhausen, Germany) in 100 µl complete medium per well in the presence of one of the following antigens: PT/FHA/PRN/FIM 2/3 (all provided by Sanofi Pasteur Inc., Swiftwater, PA, USA) or tetanus- plus diphtheria-toxoid (DT) (Chiron Behring GmbH, Liederbach, Germany). The following optimized antigen concentrations were added [6]: PT 2 µg/ml (heat-inactivated at 80°C for 20 min), FHA 10 µg/ml, PRN 10 µg/ml, FIM 2/3 10 µg/ml, and DT 7.22 Lf/ml each. Cultures containing medium without antigens were negative controls. Because of limited numbers of PBMC available, not all of the individuals were tested for each antigen. Cell cultures were performed in a humidified atmosphere at 37°C and 5% CO_2_.

### Immunophenotyping

Immunophenotyping of peripheral blood lymphocytes was performed using anti-human monoclonal antibodies (FITC anti-human CD69, PE-Cy5 anti-human CD4, APC anti-human CD8, all BD Biosciences Pharmingen™, San Jose, USA).

After 48 h of culture with specific antigens, lymphocytes were incubated for 30 min in the dark with 3 µl of each antibody in FACS tubes (5 ml round-bottom tubes, BD Falcon). The cells were washed with 2.5 ml PBS (phosphate buffered saline, Dulbecco 1*, LE, Biochrom AG) and analyzed on a FACSCalibur flow cytometer (Becton Dickinson, NJ, USA) with data stored in list mode files. Ten thousand cells were analyzed for each sample. Isotype- and fluorochrome- matched control MAb were used to determine nonspecific background binding.

### Cytokine measurement

To determine pertussis-specific cytokine production, 90 µl of supernatant of lymphocyte stimulation cultures were harvested after 48 hours, quick-frozen and stored at −20°C. The “Human Th1/Th2 cytometric bead array-Kit” (BD Biosciences, San Jose, USA) was used for the analysis according to manufacturer's instructions. Results were obtained with BD CBA Biosciences software (BD Biosciences, San Jose, USA). Cytokine quantification was performed for representative Th1 (IL-2, TNF-α, INF-γ) and Th2 (IL-4, IL-5, IL-10) cytokines.

### Data analysis and statistical methods

Data were analyzed with GraphPad Prism version 4.01 for Windows (GraphPad Software, San Diego, California, USA). Wilcoxon matched-pair signed-rank test for non-parametric data was used for analysis of pre- versus post-vaccination values for each individual. For the pairwise testing between the three groups, a Mann-Whitney U Test for non-parametric data was performed. For all analyses, a two-tailed P-value ≤0.05 was considered to be significant.

## Results

### Demographic characteristics of participants

The study participants (n = 78; 5aP group  = 37; 4aP group  = 23; 4wcP group  = 18) were comprised of 33 girls and 45 boys. The mean age of study participants was 12.4 years (age range 10.0–14.0 years). There were no substantial gender or age differences between the three groups.

### Immunophenotyping of lymphocyte activation

Immunophenotyping of lymphocyte differentiation and activation markers was performed with antibodies against CD4, CD8, and CD69 in the antigen-stimulated cultures before and 4 weeks after vaccination.

**Figure 1:**
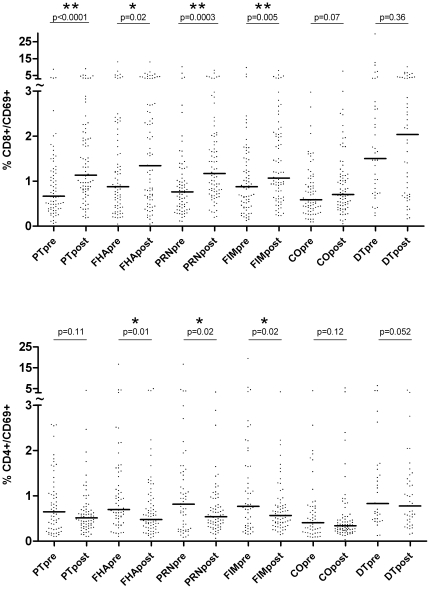
CD69^+^ antigen specific T cells after aP booster vaccination. Surface markers on T-lymphocytes were measured after 48 h of stimulation with pertussis antigens (PT, FHA, PRN, FIM), without antigen stimulation (CO), or with diphtheria/tetanus (DT) antigen stimulation. Immunophenotyping was performed prior to (pre) and 28–36 days after (post) aP booster vaccination. The percentages of CD8^+^CD69^+^ **(a)** and CD4^+^CD69^+^ **(b)** lymphocytes within the total lymphocyte population are shown for all participants. To show outliers, the y-axis was divided into two scales (tilde). Scatter blot; horizontal lines indicate the median value. * significant increase/decrease after vaccination (Wilcoxon-test; p<0.05). ** highly significant increase/decrease after vaccination (Wilcoxon-test; p<0.01).

CD8^+^CD69^+^ cells in antigen stimulated cultures were more frequent after vaccination than before (figure 1). This increase after vaccination was significant for stimulation with all four tested pertussis antigens (Wilcoxon test; PT: median increased from 0.67% to 1.14%, *P*<0.0001; FHA: median increased from 0.88% to 1.35%, *P* = 0.0209; PRN: median increased from 0.76% to 1.17%, *P* = 0.0003; FIM: median increased from 0.88% to 1.07%, *P* = 0.0054). CD8^+^CD69^+^ activated lymphocytes increased in all three groups independent of the different primary vaccination schedules. Proportions of CD8^+^CD69^+^ cells did not change significantly in DT stimulated or unstimulated control cultures.

CD4^+^CD69^+^ activated lymphocytes did not change after vaccination in PT stimulated cultures and decreased in FHA (median decreased from 0.70% to 0.48%, *P* = 0.0141), in PRN (median decreased from 0.82% to 0.54%, *P* = 0.0195) and FIM (median decreased from 0.77% to 0.57%, *P* = 0.019) stimulated cultures. There was again no significant change in DT stimulated or unstimulated control cultures (figure 1).

### Cytokine response

**Figure 2:**
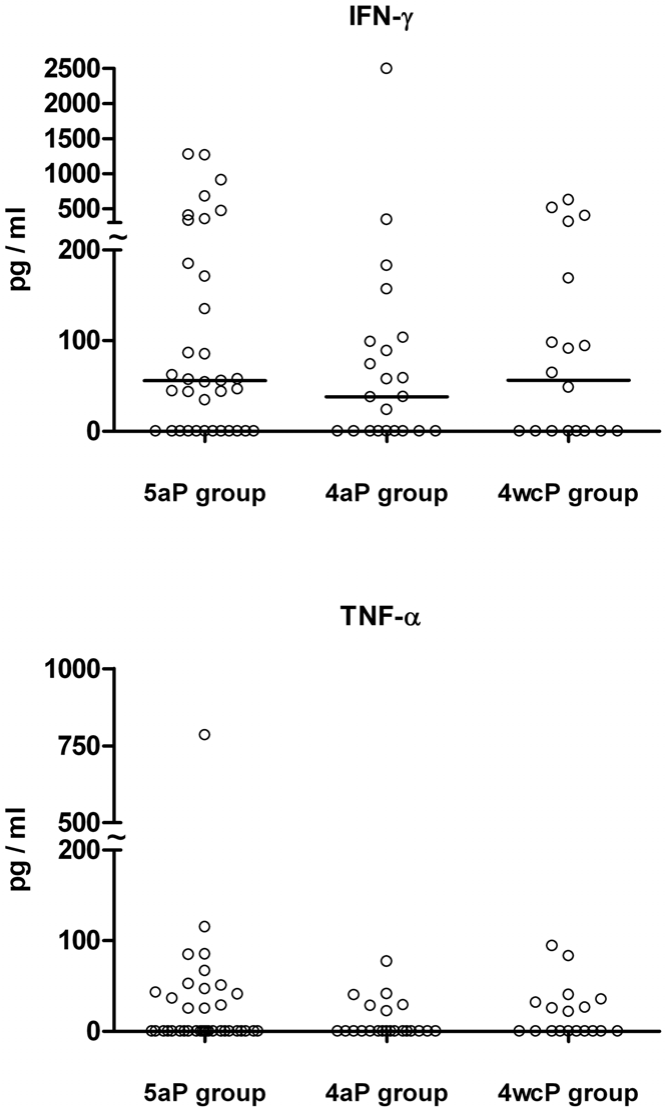
IFN-γ and TNF-α secretion after aP booster vaccination. Cytokine secretion in post-booster culture supernatants was measured after 48 h of stimulation with PT by cytometric bead arrays. To show outliers the y-axis was divided into two scales (tilde). Horizontal lines indicate the median value.

Cytokines indicative of a Th1 response (IL-2, TNF-α, INF-γ) and Th2 response (IL-4, IL-5, IL-10) were determined in supernatants of PT-stimulated cultures after vaccination. From these cytokines only INF-γ and TNF-α were above the level of detection (20 pg/ml) in a sufficient number of participants (49/78 (63%) and 29/78 (37%), respectively) to allow valid statistical evaluation between the three groups. Scatter blots of the individual INF-γ and TNF-α values upon PT-stimulation in the three groups are shown in figure 2. There were no significant differences between the three groups in the INF-γ and TNF-α secretion upon PT-Stimulation (Mann-Whitney U test).

A Th2 cytokine secretion (IL-4, IL-5, IL-10) was seen in 11/78 (14%) participants. Table 1 summarizes the distribution between the three groups. There were no detectable Th2 cytokine responses in participants with exclusive wcP preimmunization.

**Table 1:** Distribution of participants and of Th1/Th2 cytokine secretion.

Group	Number ofparticipants	One or more Th1 cytokine(s) above detection level(IL-2, TNF-α, INF-γ)	One or more Th2 cytokine(s) above detection level(IL-4, IL-5, IL-10)
**5aP group**	37	28 (76%)	9 (24%)
**4aP group**	23	15 (65%)	2 (9%)
**4wcP group**	18	16 (89%)	0 (0%)

The distribution of all participants and of the participants with detectable Th1/Th2 cytokine secretion within the three vaccination groups is shown. There was no Th2 cytokine secretion detectable in participants with exclusive wcP preimmunization.

### Th2 response and reactogenicity to vaccination

A Th2 response to DTaP vaccination has been associated with large local reactions such as extensive swelling of the whole limb [15]. Information on the reactogenicity after vaccination exists for nine of the eleven subjects with measurable Th2 cytokine secretion (simultaneous participation in the reactogenicity study [12]). One of these nine vaccinees showed large swelling and erythema, whereas the remaining eight did not develop any swelling or erythema. Taken together, the results of the nine vaccinees did not show an apparently stronger reactogenicity (fever, pain, erythema, swelling) than the general study population [12].

## Discussion

Our study focused on the CD4^+^ helper cell and CD8^+^ cytotoxic cell response in adolescents receiving an aP booster immunization after whole cell or acellular primary vaccination. Here we show that the adolescent aP booster vaccination in our study population predominantly leads to a Th1 immune response, irrespective of a whole cell or acellular primary vaccination. The vaccination also induced a significant increase in CD8^+^CD69^+^ T cells responsive to pertussis antigens. This study is the first to demonstrate CD8^+^ memory T cell immune responses to pertussis vaccine.

Four weeks after the adolescent aP vaccination CD8^+^CD69^+^ activated T cells were augmented when PBMC were stimulated with all four tested pertussis antigens, whereas CD4^+^CD69^+^ T cells were decreased in FHA, PRN and FIM stimulated cultures. There were no changes of proportions of CD8^+^CD69^+^ T cells or CD4^+^CD69^+^ T cells in control cultures. These results suggest a targeted activation of pertussis specific CD8^+^ T cells after vaccination. As expected for the quantification of antigen specific T cells in peripheral blood, the percentage of T cells specifically activated by pertussis antigens was generally low. A similar percentage has recently also been shown for proliferating CD3^+^ blasts in PT stimulated PBMC cultures after primary wcP vaccination [16].

Priming of memory CD8^+^ T cells has been described for immunizations against several intracellular pathogens, particularly viruses (e.g. encephalomyocarditis virus (EMCV), lymphocytic choriomeningitis virus (LCMV), varizella zoster virus (VZV), hepatitis B virus (HBV)) [17]–[23]. Memory CD8^+^ T cells become more rapidly induced to express activation markers or cytokine receptors and to secrete higher levels of cytokines such as IFN-gamma and IL-2 than naïve T cells. Such differences in immune responses between naïve and memory CD8^+^ T cells after vaccination have already been described for the expression of CD69 and IL2-receptor alpha chain (CD25) in humans [17], [18], [21] and the secretion of IL-2 and INF-gamma in mice [19], [20]. These differences may lead to lower activation thresholds in primed memory CD8^+^ T cells compared to naïve CD8^+^ T cells and might play an important role in the cellular memory response after vaccination, especially against facultative intracellular pathogens like *B. pertussis* [24]. Such an important role for cytotoxic T cells could at least partially explain why a protective antibody titer against pertussis infection cannot be defined [25]. In mice, however, clearance of *B. pertussis* from the lungs was mediated by pertussis specific CD4^+^, but not by CD8^+^ T cells [26]. The role for cytotoxic memory CD8^+^ T cells in protecting humans from clinical pertussis cannot be determined by our study.

Regarding cytokine secretion, we have shown here that the adolescent aP booster vaccination predominantly leads to a Th1 immune response, irrespective of a previous whole cell or acellular primary vaccination. Th1 predominant immune responses have been observed after natural pertussis infections and wcP vaccinations, whereas Th2-skewed immune responses were seen after acellular pertussis vaccinations [6]–[9]. These results were based on studies in infants and young children after their primary aP vaccination. Among adolescents and adults from the APERT study with either previous wcP or without previous pertussis vaccination an aP dose led to a predominant Th1 response [27]. In our study only a few participants of the aP preimmunization groups showed a Th2 cytokine secretion *in vitro*. In the vast majority the adolescent aP booster primed T cells to a Th1 response even in the aP preimmunization groups. It can be speculated that this might indicate previous exposure to *B. pertussis* without development of typical symptoms, so called ‘silent’ boosting. Ausiello et al. have already demonstrated the switch from a predominant Th2 immune response after acellular primary vaccination in the first year of life to a predominant Th1 immune response in 4 year old children before booster vaccination [10]. The most probable explanation to this phenomenon in this longitudinal observation was natural exposure to *B. pertussis* and a high rate of subclinical natural boosters. Studies on antibody seroprevalence and cell-mediated immunity (CMI) in our adolescent vaccinees also pointed to an influence of subclinical natural boosters on pertussis specific immunity [11]. This hypothesis is in line with data from Yano et al., who describe that in adults who were once primed for a Th1-biased immune response by wcP vaccine or by natural pertussis infection, the pertussis specific T cell pool maintains their Th1-biased responses even when boosted with aP vaccine [28].

A vigorous Th2 response after acellular pertussis vaccination in infancy and early childhood is associated with large local reactions like extensive swelling of the whole limb [15]. In our study, of 9 adolescents with measurable Th2 responses and available reactogenicity data [12] one had a large local reaction. With respect to the overall low number of participants with Th2 responses this does not clearly argue for or against that association. Our 10–14 year old study population showed a markedly lower rate of local reactions (swelling and redness at the injection site), especially of large local reactions (redness/swelling >10 cm) [12], than at the 4–6 year booster vaccination [14]. This decrease in local reactogenicity within the two studies might primarily be due to the use of an antigen-reduced acellular pertussis vaccine in adolescents. A switch to a predominant Th1 response after subclinical natural booster infections in the meantime could play an additional role for the lower local reactogenicity.

In summary, this study shows that the adolescent aP booster vaccination leads to a Th1 and CD8^+^ memory T cell immune response, which may contribute to a positive safety and efficacy profile.
